# British Congress on Tuberculosis

**Published:** 1901-07-27

**Authors:** 


### Hospital Clinics and Medical Progress.

### BRITISH CONGRESS ON TUBERCULOSIS.

THE Congress on Tuberculosis was formally inaugurated at St. James's Hall on Monday afternoon by the Duke of Cambridge. The proceedings were marked with great cordiality. After calling upon the hon. secretary general (Mr. Malcolm Morris) to read his report, the Duke of Cambridge welcomed the congressists in the name of the King. After this many speeches were made by delegates and others from many countries. On Tuesday the event of the day was Professor Koch's address, while on Wednesday Professor Brouardel, of Paris, addressed the Congress. The social arrangements for the welcome of the delegates have been on an elaborate scale.

### PROFESSOR KOCH'S ADDRESS.

By far the most important point raised by Professor Koch in the address which he delivered on Tuesday in St. James's Hall was in regard to the identity, or rather the non-identity, of human and bovine tuberculosis; for if he is right in this matter a great deal of what we are being urged to do for the suppression of tuberculosis in cattle is beside the mark, and the money which we are asked to expend in compensation for the destruction of infected animals might be spent with far more effect upon measures having for their object the breaking up of that vicious circle of infection, by way of dust and sputum, which we know to be at the main route by which the disease is spread. Professor Koch began by going over the old ground in regard to the infective nature of tuberculosis.

"Strictly speaking," he said, "the fact that tuberculosis is a preventible disease ought to have become clear as soon as the tubercle-bacillus was discovered, and the properties of this parasite and the manner of its transmission became known. I may add that I, for my part, was aware of the full significance of this discovery from the first, and so will everybody have been who had convinced himself of the causal relation between tuberculosis and the tubercle-bacillus."

The question is *how* to prevent it, and in regard to this the first and most important lesson we have learned from the success which has recently been achieved in dealing with parasitic diseases is that it is a great blunder to treat pestilences uniformly. This was done in former times; no matter whether the pestilence was cholera, plague, or leprosy: isolation, quarantine, useless disinfection, were always resorted to. But now we know that every disease must be treated according to its own special individuality, and that the measures to be taken against it must be most accurately adapted to its special nature, to its etiology. We are entitled to hope for success in combating tuberculosis only if we keep this lesson constantly in view.

Take as an example plague. People used to act upon the conviction that a plague patient was in the highest degree a centre of infection, and that the disease was transmitted only by plague patients and their belongings. Even the most recent international agreements are based on this conviction. Although, as compared with formerly, we now have the great advantage that we can, with the aid of the microscope and of experiments on animals, recognise every case of plague with absolute certainty, and although the prescribed inspection of ships, quarantine, the isolation of patients, the disinfection of infected dwellings and ships, are carried out with the utmost care, the plague has, nevertheless, been transmitted everywhere, and has in not a few places assumed grave dimensions. Why this has happened we know very well, owing to the experience quite recently gained as to the manner in which the plague is transmitted. It has been discovered that the real transmitters of the plague are the rats. There is no longer any doubt that, in by far the majority of the cases, in which the plague has been transmitted by ocean traffic, the transmission took place by means of plague among the ship rats. It has also been found that, wherever the rats were intentionally or unintentionally exterminated, the plague rapidly disappeared; whereas at other places where too little attention had been paid to the rat plague, the pestilence continued. Thus to protect ourselves from plague we must attend to the rats rather than to general quarantine.

Going on to instance cholera, hydrophobia, and leprosy, Professor Koch showed that each disease has its own manner of diffusion and that only by adapting our measures to the habits of such diseases are we likely to be successful in their suppression. Thus we come to the great question whether what has hitherto been done, and what is about to be done against tuberculosis, really strikes at the root of tuberculosis, meaning, of course, by the term tuberculosis only those morbid conditions which are caused by the tubercle-bacillus.

In by far the majority of cases of tuberculosis the disease has its seat in the lungs, and has also begun there. From this fact it is justly concluded that the germs of the disease, *i.e.*, the tubercle-bacilli, must have got into the lungs by inhalation. As to the question where the inhaled tubercle-bacilli have come from, there is also no doubt. On the contrary, we know with certainty that they get into the air with the sputum of consumptive patients. This sputum, especially in advanced stages of the disease, almost always contains tubercle-bacilli, sometimes in incredible quantities. By coughing, and even speaking, it is flung into the air in little drops, *i.e.*, in a moist condition, and can at once infect persons who happen to be near the coughers. But then it may also be pulverised when dried, in the linen or on the floor, for instance, and get into the air in the form of dust.

In this manner a complete circle, a so-called *circulus vitiosus*, has been formed for the process of infection, from the diseased lung, which produces phlegm and pus containing tubercle-bacilli, to the formation of moist and dry particles (which, in virtue of their smallness, can keep floating a good while in the air), and finally to new infection, if particles penetrate with the air into a healthy lung and originate the disease anew. Just, then, as rats in plague, and water in cholera, so dust and sputum are the things to pay attention to in the prevention of consumption.

But another possibility of tuberculous infection exists, as is generally assumed, in the transmission of the germs of the disease from tuberculous animal to man. This manner of infection is generally regarded nowadays as proved, and as so frequent that it is even looked upon by not a few as the most important; and the most rigorous measures are demanded against it. Professor Koch says, however, that his investigations have led him to form a different opinion. Genuine tuberculosis has been observed, he says, in almost all domestic animals, and most frequently in poultry and cattle. The tuberculosis of poultry, however, differs so much from human tuberculosis that we may leave it out of account as a possible source of infection for man. So, strictly speaking, the only kind of animal tuberculosis remaining to be considered is the tuberculosis of cattle, which, if really transferable to man, would indeed have frequent opportunities of infecting human beings through the drinking of the milk and the eating of the flesh of diseased animals. Even in his first circumstantial publication on the etiology of tuberculosis Professor Koch expressed himself with reserve regarding the identity of human and bovine tuberculosis. But later experience had enabled him to speak with increased confidence. He related various instances in which young cattle, which had stood the tuberculin test, and might therefore be regarded as free from tuberculosis, were infected in various ways with pure cultures of tubercle bacilli taken from cases of human tuberculosis, and yet none of them showed any signs of disease, and after they were killed not a trace of tuberculosis was found in their internal organs. Only at the places where the injection had been made small suppurative foci had been formed in which a few tubercle bacilli could be found. But on infecting cattle with cultures derived from an animal suffering from bovine tuberculosis the results were quite different, the cattle proving just as susceptible to the bacillus of bovine as they had proved insusceptible to that of human tuberculosis. Other experiments were detailed which he held to justify him in maintaining that human tuberculosis differs from bovine and cannot be transmitted to cattle. But how is it with the susceptibility of man to bovine tuberculosis? To this question it is impossible to give a direct answer, because experimental investigation is, of course, out of the question.

It is well known, however, that the milk and butter consumed in great cities very often contain large quantities of the bacilli of bovine tuberculosis in a living condition, as numerous experiments with such dairy products on animals have proved. Most of the inhabitants of such cities daily consume such living and perfectly virulent bacilli of bovine tuberculosis, and unintentionally carry out the experiment which we are not at liberty to make. If the bacilli of bovine tuberculosis were able to infect human beings, many cases of tuberculosis caused by the consumption of alimenta containing tubercle bacilli could not but occur among the inhabitants of great cities, especially the children. And most medical men believe that this is actually the case.

In reality, however, it is not so. That a case of tuberculosis has been caused by alimenta can be assumed with certainty only when the intestine suffers first—*i.e.*, when a so-called primary tuberculosis of the intestine is found. But such cases are extremely rare, and it is just as likely that such cases as do occur have been caused by the widely propagated bacilli of human tuberculosis, which may have got into the digestive canal in some way or other. Hitherto no one could decide whether such cases were of human or animal origin. Now, however, we can diagnose them. All we have to do is to inoculate a pure culture into cattle, and if bovine they will "take," if human they will not. Such results as Professor Koch has been able to obtain from this proceeding "do not speak for the assumption that bovine tuberculosis occurs in man."

Though the important question whether man is susceptible to bovine tuberculosis at all is not yet absolutely decided, and will not admit of absolute decision to-day or to-morrow, one is nevertheless already at liberty to say that, if such a susceptibility really exists, the infection of human beings is but a very rare occurrence. "I should estimate the extent of infection by the milk and flesh of tuberculous cattle, and the butter made of their milk, as hardly greater than that of hereditary transmission, and I therefore," said Professor Koch, "do not deem it advisable to take any measures against it."

The only main source of the infection of tuberculosis is then the sputum of consumptive patients, and the measures for the combating of tuberculosis must aim at the prevention of the dangers arising from its diffusion.

The Chairman (Lord Lister) in thanking Professor Koch for his address, which was, he said, full of profound interest from beginning to end, pointed out that while, if Professor Koch were right in regard to the relation between human and bovine tuberculosis, prevention would be greatly simplified, it would be a grievous matter if the efforts now being made to secure purity of milk and meat supplies should be relaxed and then it should turn out that, after all, bovine tuberculosis was dangerous to man.

Professor Nocard, speaking as a member of the veterinary profession, said that the facts related by Professor Koch might be explained by the modification which microbes undergo from their environment, and in regard to incommunicability from animals to man he stated that veterinary surgeons were not infrequently inoculated from bovine disease by accident, and that there were many other examples of similar inoculation.

Professor Bang, of Copenhagen, thought that Professor Koch had gone a little too far in saying that there was no necessity for taking measures against bovine tuberculosis.

Professor Sims Woodhead, of Cambridge, said he was convinced that bovine tuberculosis did play some part in the extension of the disease. He hoped that the Minister of Agriculture would institute experiments by which the point might be decided, but for the present he strongly urged that they should continue to take precautions.

### Professor Brouardel's Address.

On Wednesday afternoon Professor Brouardel, addressing a large audience in St. James' Hall said:—Mr. President, Ladies and Gentlemen, mortality from tuberculosis varies according to the country. In some cases it is accountable for a sixth, a fifth, and sometimes a fourth of the total mortality. Havoc such as this makes it compulsory that all nations and governments should strictly inquire into, and adopt, measures to arrest the propagation of a disease which, in these days, is the greatest enemy of the human race.

The disease used to be considered incurable; but when, on December 5th, 1865, Villemin showed experiments at the Academy of Medicine, which proved the real presence of the contagion, and when our illustrious colleague, Professor Robert Koch, had discovered and demonstrated to the medical world the agent of this contagion, every one felt that a new way was opened to humanity, and every nation now wishes to profit for the public good by the recent scientific discoveries.

I was asked by the Committee to make the subject of my address the methods adopted by different nations for the prevention of consumption. But, gentlemen, the grounds for the prevention of tuberculosis are identical in every country. On this question the entire medical profession of the world is united.

Tuberculosis is avoidable and curable.

The methods used to bring this about are the same in all countries, but according to the impetus given to the movement by the scientists in the different nations, and according to the customs and laws in force, some nations have taken the first rank.

It is only possible to bring a law into force that interferes with our daily life, that disturbs inveterate habits, and that has to be carried out in the bosom of the domestic hearth, when it is called for by public opinion: when all are convinced of its benefits, and every one recognises the danger of his vicious habits, and is ready personally to reform them and to require his neighbour to do the same.

The first thing which is indispensable is to educate public opinion. Gradually in all countries the public are beginning to realise that personal care and cleanliness are necessary to obviate contagion, and are also realising that other idea, to my mind equally important, that a consumptive patient is only dangerous if the necessary precautions are not taken around him, and if he himself does not take them to protect his relatives, friends, and fellow-workmen from contagion.

The danger is in the sputum, which contains thousands of contagious germs. To expectorate on the ground is a disgusting and dangerous habit. Once this habit has quite disappeared, tuberculosis will decrease rapidly.

What *rôle,* as a matter of fact, does this sputum play in the subsequent propagation of the disease? It varies in different cases.

Collected and shut up in a private, or common but antiseptic, spittoon, destroyed by incineration or some other measure, it is dangerous to no one.

Thrown into dry and well-lighted surroundings, exposed to the rays of the sun, it will soon lose its dangerous properties.

But if it remains in damp and dark surroundings it will maintain its activity for a long time.

Thus it is that tuberculosis claims more victims from gloomy, ill-ventilated, dark dwellings.

Unhealthy dwellings, however, cause other disasters. Dark and crowded as they are, cleanliness is difficult, if not impossible, to preserve; they are not pleasant to pass the time in, and the workman stays in his home as little as possible; he eats there and sleeps there, but the rest of his time is spent in the public-house. J. Simon was right in saying: "The wretched lodging is the purveyor of the public-house," and we can add to it that the public-house is the purveyor of tuberculosis.

In fact, alcoholism is the most potent factor in propagating tuberculosis. The strongest man who has once taken to drink is powerless against it. A universal cry of despair rises from the whole universe at the sight of the disasters caused by alcoholism.

Ministers who have the charge of the financial department of the State like to calculate the sum the State gets from the duty on alcohol, but they should deduct from it the cost to the community of the family of the ruined drunkard, his degenerate, infirm, scrofulous, and epileptic children, who must have shelter.

The conditions of modern life compel a man to live in crowds. This peril from common life, inseparable from advance in civilisation, is continually growing: it is the ransom, and accounts for the threatening increase in tuberculosis.

Brought face to face with those predisposed by their family history, by the unhealthiness of their dwellings, it has been recognised as a duty in some countries to make an attempt to restore these tottering frames, and to render them capable of baffling with the dangers that threaten them, by the establishment of hospitals and sanatoria, which are now numerous.

Before touching on the question of the cure of tuberculosis I should like to say that, with no wish to exaggerate the danger of the propagation of tuberculosis by meat, it cannot be overlooked. It is easy, by means of legislation, to protect the population from this method of contamination. Belgium has set us the example.

That the milk of cows with tuberculous inflammation of breast is used is very clear. Your great hygienist, Dr. Thorne-Thorne, while pointing out that in England mortality in general of adults from phthisis has diminished since 1850 from 45 per hundred, regrets to see that from the same date infantile mortality from tuberculosis increased by 27 per hundred. According to him, this increase is entirely due to tuberculosis in the abdomen, caused by ingestion of contaminated milk in infants under a year old. It is well to add that in large concerns the milk from different sources is mixed, and one cow only need be the victim of tuberculous mammitis in order to contaminate all the milk with which its milk is mixed. To prevent this method of propagation, strict inspection measures should be adopted, such as have been in use for several years in Denmark, Sweden, and Norway, to the great advantage of public health.

If a man is the victim of tuberculosis, everything possible should be done to cure him, for *he can be cured.* There is no doubt of this, and everything possible must therefore be done to bring this about by careful organisation. After referring to the compulsory notification and sanatorium treatment as means for the prevention of consumption, Professor Brouardel ended by an eloquent peroration in favour of all joining in the common endeavour to stamp out the cruelest scourge that has ever fallen upon us.

